# An Untapped Potential in Primary Care: Semi-Structured Interviews with Clinicians on How Patient Portals Will Work for Caregivers in the Safety Net

**DOI:** 10.2196/18466

**Published:** 2020-07-20

**Authors:** Alejandra Casillas, Anupama Gunshekar Cemballi, Anshu Abhat, Miya Lemberg, Jennifer D Portz, Shobha Sadasivaiah, Neda Ratanawongsa, Wagahta Semere, Arleen Brown, Courtney Rees Lyles

**Affiliations:** 1 Division of General Internal Medicine and Health Services Research Department of Medicine UCLA David Geffen School of Medicine Los Angeles, CA United States; 2 UCSF Division of General Internal Medicine at Zuckerberg San Francisco General Hospital UCSF Center for Vulnerable Populations San Francisco, CA United States; 3 Los Angeles County Department of Health Services Harbor-UCLA Medical Center Los Angeles, CA United States; 4 Division of General Internal Medicine University of Colorado School of Medicine Aurora, CO United States; 5 Office of Health Informatics San Francisco Health Network San Francisco, CA United States

**Keywords:** patient portal, caregivers, vulnerable populations, digital divide, mobile phone

## Abstract

**Background:**

Patients within safety-net settings are less likely to access health information on patient portals, despite expressed interest. Family and friends are important resources to assist these patients (ie, Medicaid recipients, older patients, patients with limited English proficiency) in navigating health systems, and provider support of the use of patient portals among these groups may also facilitate caregivers’ use of their patients’ portal.

**Objective:**

Because safety net providers work closely with caregivers to care for their patients, we used qualitative methods to explore safety net providers’ perspectives on portal use among caregivers for their patients, especially as there is limited literature about caregivers’ use of portals in the safety net.

**Methods:**

We conducted 45- to 60-min semistructured telephone interviews with providers from three large California safety-net health systems. The interviews focused on providers’ experiences with caregivers, caregiver roles, and how the portal could be leveraged as a tool to support caregivers in their responsibilities. A total of three coders analyzed the interview transcripts using both deductive and inductive approaches and established a consensus regarding major themes.

**Results:**

Of the 16 participants interviewed, 4 specialized in geriatrics, and all held a leadership or administrative role. We described themes highlighting providers’ recognition of potential benefits associated with caregiver portal use and specific challenges to caregiver engagement.

**Conclusions:**

Providers recognized the potential for portals to improve information delivery and communication by helping caregivers assist socially and medically complex patients in the safety net. Providers in safety net sites also discussed a clear need for better ways to keep in touch with patients and connect with caregivers, yet security and privacy are perhaps of higher importance in these settings and may pose challenges to portal adoption. They noted that caregivers of patients in the safety net likely face similar communication barriers as patients, especially with respect to digital literacy, health literacy, and English proficiency. Further research is needed to assess and support caregivers’ interest and ability to access portals across barriers in health and digital literacy, and English proficiency. Portal platforms and health systems must also address specific strategies to uphold patient preferences while maintaining privacy and security.

## Introduction

### Background

Growing evidence on the benefits of patient engagement has fueled health systems’ focus on patient portals as a central access point for the future of primary care [1,2]. The financial incentives of the Meaningful Use Program, as part of the US health care reform in 2014, spurred a rapid uptake of patient portals across health systems nationwide [3-5]. Emerging national evidence in the last 5 years indicates that the digital divide is shrinking, as 80% of the US population owns internet-accessible smartphones, without differences by race or ethnicity and with few differences across income categories nationally, especially when it comes to mobile use [6,7].

Despite improved health information technology (IT) access, there is a large body of evidence demonstrating significantly lower use of digital health care tools among underserved populations, underscoring the need for more research to understand the contextual factors affecting their use [8,9]. Vulnerable patients within safety-net settings (eg, low socioeconomic status, under- or uninsured, limited English proficient [LEP], aging, and physically or mentally disabled) are interested in accessing their health information on patient portals but are less likely to actually do so. Numerous studies have shown that there is a high level of patient interest in portals, repeatedly documented among LEP populations, Medicaid/Medicare recipients, and older patients [10-14]. A 2014 survey of Spanish-speaking patients in a Los Angeles Federally Qualified Health Center found that the majority of patients had computer access (66%), internet access (78%), a current email account (78%), or a smartphone (71%) and that 75% of patients were interested in using a patient portal [10].

Family and friends are important resources to assist these underserved populations (especially those that are Medicaid recipients, older patients, or LEP) in navigating health systems, and provider support could facilitate their portal engagement. Caregiver engagement with a patient’s portal may range from simply assisting the patient to log on and use the portal to logging onto a patient portal for the patient (unofficial patient *surrogate*) all the way to acting as a registered proxy for portal access (where caregivers are granted their own registered access to a patient’s health information on the patient portal with the patient’s permission) [15,16]. Older patients, especially those who are LEP, tend to navigate health system processes with family members or close friends who schedule and attend medical visits, coordinate care, manage medications, assist with self-care tasks, and facilitate transitions across care settings [15,16]. Patients with low health literacy similarly benefit from the involvement of trusted family or friends during medical visits [17,18]. Prior studies among older patients have confirmed that family members facilitate patient access and use of the patient portal [19,20] and utilize the patient portal for the patient [21-25]. Given that these patients are interested in sharing electronic health information with family members or close friends [19,20,26,27], tailored strategies are needed to engage family members or friends in accessing health care digitally, especially in safety-net settings with a majority proportion of vulnerable patients and where caregivers play a key role in patient care.

The growing role of caregivers and caregiver/proxy portal use among vulnerable patients presents a potential well-aligned digital strategy for reducing racial/ethnic disparities in health that can be facilitated via already available health IT. However, buy-in from physicians and other health care providers will be important in promoting patient portals on this next level, especially in safety-net settings [28,29]. The aim of this qualitative study was to explore safety-net providers’ perspectives on caregiver/proxy portal use.

## Methods

### Design and Study Setting

The study was approved by the University of California, San Francisco and University of California, Los Angeles institutional review boards. For this descriptive, qualitative study [30], we conducted individual telephone semistructured interviews with a purposive sample of physicians or providers (n=17), working in a California safety-net setting in Los Angeles, San Francisco, or Alameda County. Los Angeles participants were recruited from the Los Angeles County Department of Health Services (LAC DHS) system. LAC DHS forms the core of the health care safety-net for indigent populations in Los Angeles County, the largest, most ethnically diverse county in the United States. LAC DHS serves more than 10 million residents and provides over two and a half million ambulatory visits every year [31-33]. In northern California, providers were recruited from the San Francisco Health Network (SFHN) and the Alameda Health System (AHS). The AHS is the East Bay’s (Oakland area) public health system with 5 hospitals and 4 primary care clinics, whereas SFHN is San Francisco City’s and county’s public health care system, providing primary care at 14 clinics, youth-focused care at 11 clinics, and care at a hospital serving over 1 million residents. The internet patient portals for the safety-net health systems included in the study allow patients to view clinical data (laboratories and radiology reports), refill medications, request/change appointments, message their health care teams, read about their prior and current diagnoses (links to educational content), and view/download their medical records.

### Participants and Recruitment

From April to November 2018, we recruited and interviewed providers from the LAC DHS, SFHN, and AHS. Providers were sought if they had clinical activity in a safety-net health setting and/or held a leadership role in any safety-net clinical setting. Los Angeles participants were recruited via personal recommendations from the medical director of digital health services in LAC DHS. The SFHN and the AHS providers were recruited via recommendation from study coinvestigators. We conducted 45- to 60-min semistructured interviews with health providers from these 3 large California safety-net health systems. Participants were interviewed via telephone. AC, AGC, WS, and CL conducted the interviews on multiple days until at least three providers were interviewed from each of the 3 health systems. Participants were provided with a US $25 gift card after completing their scheduled interviews.

### Data Collection

The semistructured interviews focused on providers’ experiences with caregivers, the roles caregivers play, and how the portal could be leveraged as a tool to support caregivers in their responsibilities. The discussion questions were modified from projects led by study investigators who examined barriers and facilitators to portal use to develop a randomized trial on patient portal training [34]. This prior research highlighted the limited knowledge and understanding of caregivers’ use of the patient portal.

Discussion introductions included participants’ self-describing clinical and administrative roles. Participants were then asked about their experiences with patient caregivers (definition, types of caregivers, and positive and challenging interactions). They were subsequently tasked to describe strategies that they have observed caregivers take on with patient health management. We asked participants about how the patient portal affects these caregiver strategies, what they thought was useful, and how they envisioned the portal could affect these patients’ and caregivers’ care. The discussions wrapped up with a conversation about what the health system could do to help improve caregiver and patient engagement with the portal.

### Analysis

Interview discussions were audio recorded and transcribed, reviewed for accuracy, and deidentified. AC and AGC independently read and summarized 6 transcripts over multiple theme meetings, applying inductive and deductive methods to identify the spectrum of themes encountered for each of the interviews with accompanying example quotations. AC and AGC then cross-referenced their independently developed list of themes. Through codebook discussions, they reached a consensus for a more final codebook. AC and AGC then coded 3 transcripts independently with this codebook for validity. The definition for each theme (code), with the final example quote, was developed by AC, with iterative feedback from the AGC and the entire group, until a clear consensus was achieved. Using the established codebook and definitions, a third coder (ML) analyzed the transcripts using Dedoose version 8.2.14. AC and AGC reviewed the analysis by ML and established a consensus regarding any other discrepancies in themes and corresponding quotes [35].

## Results

### Participant Characteristics and Theme Categories

Of the 16 participants interviewed, 4 specialized in geriatrics, and all held a leadership or administrative roles, as shown in Table 1. All the participants worked in primary care settings, including a speech language pathologist.

We described primary themes highlighting safety-net physicians’ and providers’ recognition of potential strong benefits associated with caregiver portal use and specific barriers to caregiver engagement. The 4 major themes are separated into 2 large categories: (1) positive aspects of portal use by caregivers (Table 2); and (2) challenges to address for portal use by caregivers (Table 3). These show major themes, subthemes, frequencies, and quotes.

**Table 1:** Safety-net provider participants’ characteristics.

Clinical specialty	Safety-net role
Geriatrics	Physician, Medical Director
Geriatrics	Physician, Department Chief
Geriatrics	Physician, Director of Primary Care
Geriatrics	Physician, Outpatient Care Medical Director
Internal medicine	Physician, Clinical Lead
Internal medicine	Physician, Clinical Lead
Internal medicine	Physician, Health Care Executive
Internal medicine	Physician, Medical Director
Internal medicine	Physician, Medical Director
Internal medicine	Physician, Resident Preceptor
Internal medicine	Physician, Director of Outpatient Care
Internal medicine	Physician, Director of Primary Care Quality
Family medicine	Physician, Assistant Medical Director
Family medicine	Physician, Health Care Executive
Family medicine	Physician, Health Care Executive
Occupational therapy	Speech Pathologist, Health Care Executive

**Table 2:** Themes and subthemes on positive aspects of portal use with exemplar quotes.

Themes/subthemes		Quotes
**Caregiver designation in the electronic health record (n=25)**		
		“I record it in my notes...at the first part of workflow...registration staff is collecting information about emergency contacts and alternate contacts...depending on what the patient or their caregiver says at registration, there might be something entered into the chart in that capacity.” “Sometimes, because usually it would be because the note would say, ‘Accompanied by daughter’ or ‘Discussed with daughter’, ‘Discussed with son’, ‘Discussed with caregiver’, ‘Discussed with IHSS [in home support services] worker,’ but not all the time.” “I don’t think uniformly. You’ll see it (caregiver information) in the body of the note, not always in the, social history section. And to tell you the truth, our EHR doesn’t have a good section on caregiver information...in terms of like whose number should you call? Do they have a caregiver they can call?” “[I’m] trying my best to collect the information and actually put it in the EHR so I know who they are...oftentimes, after I did that, [with] a group of patients, I always follow up if they live in supportive housing, or they have a case manager and so that’s external, nonfamily caregiver visit their office, followed up by email to whoever their support people are in the community to make sure that they know what the next steps are.”
**Caregiver use and potential use of the portal (n=55)**		
	Portal as a tool to assist caregivers with standard health care tasks (n=32)	“I think caregivers using the patient portal could co-manage patient’s health, could be a huge asset to the caregiver and the provider... it’s not infrequent that the patient or the patient and the caregiver show up to appointments saying they ran out of their meds two months ago.” “Usually there’s more than one caregiver, where there’s a network and may have varied involvement. Like with my sisters and me, we have this constant flow of information, and having a way to put it all in one place to share easily is really important.” “...they’re just rolling out this new scheduling tool that will allow internal schedulers to be able to search for ways of grouping their appointments. So, the [OT, PT,] speech could all happen on the same day. If that tool could be made available to a caregiver so they can say ‘Okay, I wanted to have doctor’s appointments at this and this and this all on the same day’, I think that would be really powerful.”
	Portal as a tool to directly support the caregiver (n=7)	“Empowering the caretakers to be able to use technology decreases their stress level.” “I think respite is also a big thing, caregiver relief and caregiver fatigue is a big problem sometimes, and having access to joint behavioral health services, or like couple counseling, or parent-child counseling in the study of chronic disease, I think it’s something super underutilized. That could probably help a lot with the challenges.” “I know there’s a lot of groups that are working on this or have published. I know at UCLA in geriatrics, they’ve done an Internet caregiving education course, and I know at Stanford, they put together Caregiving Ed. And at the VA, there’s several well published evidence-based caregiving teaching programs, but our patients certainly don’t have—or a lot of my patients don’t have access to that.” “I would be really interested in the opportunity to do more training for caregivers. I think that’s a great business opportunity, because so many caregivers have no idea what they’re doing and would be happy to get trained. You can imagine the range of trainings, but our organization or your organization, or you know some private—any big county—I could imagine offered some form of training for caregivers. I think having a portal will help hopefully and I think having processing in place to clarify what can be shared and what can’t be shared with caregivers, if it’s documented well.”
	Expanding portal functionality for caregiver use (n=18)	“For caregivers who have a homebound elder, to be able to, you know, put on their FaceTime and for me to see what’s going on that would be amazing.” “...allowing patients to search and contribute to the medical notes before the visit, the medical records, typing in their symptoms. I think there should be conversations of getting caregivers involved in the patient’s care.” “If people just keep track of hey, someone’s calling not for themselves and just kept a running list, and then you do active outreach for anyone who is calling not for themselves.”

**Table 3:** Themes and subthemes on challenges to portal use with exemplar quotes.

Themes/subthemes		Quotes	
**Portal privacy and security in the caregiver-provider-patient triad (n=38)**			
	Portal triad relationship (n=7)		“The one downfall of caregivers having access is, is there any loss in translation or, if some plan is made on the portal, is that being followed through by the caregiver?” “One of the few that I communicate with is a husband and wife patient and this is a challenge...because then I have to document it in her, in his chart, where her messages get documented in his chart... and it’s worked because she has direct access to me and then he has direct access too, but she uses her account to...manage his health... And, yeah, I thought it would be nice if she could switch back and forth.” “If it’s something I can communicate to them immediately, then I send a message through the portal. Then, if there’s anything else that I might need to communicate with them, and I don’t need to call them, then I’ll do it through the portal. A lot of it is dependent on, ‘What can I communicate briefly in writing without creating [confusion] on their part or some more questions than answers?’” “I’m observing body language, so on rare occasions, if I’m concerned that perhaps the person feels like they can’t ask the other person to leave the room, then I go ahead and...When I’m gonna be asking something sensitive in the interview around social history or any history, I’ll actually ask the other person to step out of the room and wait in the waiting room and I just normalize that and say, ‘You know, I always do this to respect confidentiality’. That’s in person, so what do you do when tech comes in?” “If you build a system that is specifically asking, ‘Is this a proxy person?’ and kind of asking for that designation right up front and putting it in black and white, you do run the risk of—of having more scrutiny and I think it’s appropriate.” “I think that what we’re doing right now is we are just in the infancy of using our portal for patient communication.” “And when I get an odd message or a little crazy message, you know, or someone requesting whatever, I screenshot it and I send it to the site managers and I tell them to call them on the phone to get more information.”
	Control of shared access (n=13)		“When we see some research that suggests that some patients don’t want the entire portal revealed to their loved ones, but maybe part of it...and so patient privacy is—is a big concern. A husband may not want his wife to know that he was a prior IV-drug user from a different life.” “I have mixed views because we try the different role for parents from the portal once the kid at 13. And sometimes they want to come for birth control and they didn’t want their moms to know. So, I don’t want the moms to see the kid’s the portal.” “I mean just mostly with like the proxy access...they’ve just had a lot of questions and concerns around, ‘When is it okay? Do we need a legal document that states, “Yes, ma’am. They are my legal healthcare proxy,”’ or if they’re, on mild dementia, but they say, ‘No, let her do it’, is that sufficient?”
	General portal security and technology concerns (n=11)		“I do have some HIV patients who won’t join because they’re just afraid of the internet, in general...I think it’s a broader concern beyond just their HIV status but just that they don’t think it’s safe to have their information in the cloud basically, because it could be stolen or, or utilized in a way that’s negative.” “How do you build a portal that respects patient privacy? You also wonder if adding a caregiver affects the security of the health data.”
**Barriers to caregiver enrollment and use (n=46)**			
			“The number of people [caregivers] who don’t have access to either a smartphone or the internet...I think is just the reflection of the income level and kind of resource constraints of our patient population.” “I’m guessing that there would be times when people are concerned by the results that look abnormal or don’t understand them because no one explained it to them....” “A lot of our patients just don’t really use like electronic technology and they don’t speak English, or they don’t have an internet connection or a computer.”

### Positive Aspects of Portal Use by Caregivers

Table 2 displays themes and subthemes for the category describing positive aspects of portal use. In the theme, *caregiver designation in the electronic health record*, participants described various potential pathways for identifying caregivers via the electronic health record (EHR) and how this would be useful. Some participants also described the challenges of not having a workflow to readily integrate current caregiver information into the EHR and how having such information available would make it easier for physicians and other providers to engage the caregivers during and outside of the clinic encounter. This was important, as the participants in the study noted multiple *caregiver types*, which are present in different ways for patients within safety-net settings: relatives, friends, case managers, in-home supportive services, and social workers.

Participants also discussed *caregiver use and potential use of the portal*, detailing current uses of patient portals by caregivers and aspirational ideas about what the patient portals could potentially do for safety-net caregivers in terms of facilitating patient care. This theme was organized into 3 subthemes: for subtheme 1, *portal as a tool to assist caregivers with standard health care tasks*, participants mentioned ways that the current versions of their patient portal supported or eased the job of the caregiver by facilitating simple health care tasks for the patient via the portal (eg, obtaining medication refills or making appointments), making it easier for the caregiver to care for the patient (as they might have to spend less time on the phone making calls and possibly decreasing the need for face-to-face visits).

For subtheme 2, *portal as a tool to directly support the caregiver*, participants discussed how portals could provide well-being/resources specifically tailored for the caregiver. For example, they mentioned that patient portals could be a way to provide explicit support to caregivers (eg, preventing caregiver burnout) and connect caregivers directly to community resources (including information about self-care and programs that assist caregivers in the safety-net) that make the caregiving job more manageable. They also noted that such resources could include a tutorial on caregiver education/navigation. The portal could provide caregivers access to tools on how to care for patients and/or how to navigate a complicated safety-net health care and social service system.

For subtheme 3, *expanding portal functionality for caregiver use*, discussions centered on a wish list of what the portal could do in the future (Portal 2.0) for caregivers. Participants mentioned how technology could help paint a better picture of the patient and caregiver via telehealth (access to home context) and more frequent communication. Such modalities could facilitate the implementation of evidence-based initiatives such as advanced care planning in the safety-net.

### Challenges to Portal Use by Caregivers

Table 3 displays themes and subthemes related to challenges to portal use by caregivers. The major theme, *portal privacy and security in the caregiver-provider-patient triad*, encompassed unresolved issues that these participants noted in communicating with the patient and caregiver through the portal. They raised several challenges that may have not been fully thought through yet as patient portals are being marketed to caregivers in the safety-net. These were summarized in 3 subthemes.

For subtheme 1, *portal triad relationship*, participants described the nuances of building or managing a relationship with the caregiver, as well as the patient, through the portal. For example, many times throughout these discussions, physicians cited the many nuanced ways in which they would validate caregiver reporting during clinic visits (talk to patient and caregiver separately), check for caregiver abuse of the patient by examining the patient or reading body language, and simply also assess how the caregiver was doing with the burden. They noted that this subtle art of relationship management and checking in with a caregiver, which are essential features of the triad relationship [24,36,37], would be very difficult to achieve via digital communication and expressed concern that some of these needed in-person checks and balances would be lost via the current version of our patient portals.

For subtheme 2, *control of shared access*, participants mentioned concerns about proxy access. For example, what if a patient allows a caregiver to log in as a proxy into their patient portal, but the patient does not want the caregiver to see their entire medical record? Or what if the proxy sends messages to the provider on the portal as the *patient*, but the provider is not aware that they are communicating with someone that is not their patient? This is an important concern in a safety-net population where patients may have sensitive diagnoses (such as sexually transmitted infections, illicit drug use, addiction, or mental illness) that have not been disclosed to the caregiver or other close friends or family because they carry some social stigma. Sensitive information on the portal could also reveal risks to family caregivers that they may not have been made aware of (genetic diseases or genetic risk factors). Participants also noted the simple workflow barriers to creating proxy login for caregivers. Although most systems have the potential to do this, most clinical settings do not outright offer it and/or facilitate the registration process.

For subtheme 3, *general portal security and technology concerns*, safety-net physicians made general comments about security or privacy issues with technology in health care. Providers worried that their patients and caregivers would not use a portal secondary to fears about their private data being accessed by outside parties (be *hacked*).

In the last major theme, *barriers to caregiver enrollment and use*, participants noted the challenges to enrolling caregivers as proxies into their patient’s portal. Many of the barriers noted paralleled the general challenges to portal use among patients in the safety-net: decreased access to and familiarity with technology (low digital health literacy), lack of easy access to the internet at home, being LEP (when most of the portal is in English), fear of signing up because of immigration documentation status, lack of awareness about a patient portal in the safety-net, or lack of understanding of what a patient portal can do.

## Discussion

### Principal Findings

Patient portals and caregiver engagement in primary care both represent clear ways to improve our communication processes with vulnerable patients by making care more convenient and coordinated. However, even in large integrated delivery systems with established track records of portal use, there is very little research on the role that caregivers can play in terms of digital communication via the EHR. Our study echoes some of the prior literature recognizing the clear need for better functionality of portals for caregivers (specific content for them and better ways for patients to designate what types of information or access to share) [22-24]. We also shed light on some specific needs in this population, such as the needed support for caregivers with communication or language barriers (which is of high importance in safety-net settings).

Beyond the health care setting, there is already a *team* around many of our patients—family members and friends who serve as caregivers and trusted confidants when making health decisions. Health leaders must recognize the need to make it easier to connect with these trusted team members, in addition to the patient, when creating care plans together, especially as systems focus on the patient portal as a primary health management tool moving forward. The next version of portals can potentially improve the integration of telehealth options into the interface and provide resources that are specific to assisting caregivers, such as local resources, to reduce or prevent caregiver burnout. These innovations may be important for patients and caregivers in safety-net settings.

Participants brought up many important privacy and communication issues to resolve and improve to make portals work for safety-net caregivers and patients. Although most safety-net EHRs have the technical capability to create proxy account functionality [38], limited information exists regarding the use of shared access. Available data indicate that health system implementation of shared access functionality has been variable [39], but national estimates are that up to 30% of portal users have used the portal as a proxy for someone else (with about 50% of them as a formal caregiver/proxy login) [40,41]. Prior studies have evaluated patients’ experiences with accessing and using their own patient portal account [21] and preferences for sharing their electronic health information with others [27,42,43]. These studies suggest that some patients would like the option of selecting the *level* at which a proxy caregiver has access to their complete medical record. As such, we found that addressing the privacy and security nuances of building a digital relationship with a caregiver in the safety-net was a major barrier to physician and provider participants being completely supportive of proxy patient portal use. In the safety-net, physicians have reported the need to provide limited portal views of *sensitive* information (eg, HIV results and prior intravenous drug use history) for patients who rely on family or friends as informal or formal caregivers. Many times, the option to use the portal is foregone by patients because of this current *all-or-nothing* access approach to patient information via a proxy login. One solution to this dilemma is to allow patients the ability to choose the level of proxy access a caregiver or family member will have. EHR vendors should be incentivized via health information policy to innovate, and safety-net health systems should advocate for more patient-centered options around *level of access* to health data via proxy relationships, especially when patient privacy and security remain a top level of concern among safety-net patients. This is a feature that should be available to all patients and will resonate with several groups, including adolescents, older adults, and patients with some sort of impairment or disability, in other health care settings.

### Limitations and Strengths

Among the limitations of this formative study are the small sample size and generalizability to other safety-net health care systems, which are different from the California settings included in the study. However, the purpose of this qualitative study is to generate initial insights about provider buy-in regarding portal potential for caregivers in the safety-net.

This is one of the few studies to probe into provider perspectives around the role of caregivers in portal use within the safety-net. We found that providers recognized the potential for portals to improve information delivery and communication, benefiting caregivers who are assisting socially and medically complex patients in the safety-net. These safety-net health care providers and leaders focused on expanding the functionality of the portal, so that it does *more* for caregivers.

### Conclusions

Overall, safety-net systems that seek to maximize the use of portals in their communities must develop specific strategies to uphold patient preferences and innovatively support caregivers while maintaining privacy and security. Further work is needed to assess and support diverse caregivers’ access to portals by addressing pivotal barriers, such as health or digital literacy and English proficiency [44]. Safety-net health systems provide health care for our most medically and socially fragile patients: populations that include patients with multiple morbid conditions, limited English proficiency, cognitive impairment, physical and mental disabilities, low literacy, homelessness, substance use disorder, justice involvement, and immigrant and refugee status. As they serve the most vulnerable, safety-nets are the ideal places to develop and refine the next patient and caregiver-centered iterations of the EHR and the patient portal. However, to make this tool work for our most vulnerable patients, we must take intentional steps to ensure that the patient portal can be effectively and efficiently deployed by their caregivers in the systems that serve these high-risk patients.

## Abbreviations

AHS: Alameda Health System
EHR: electronic health record
IT: information technology
LAC DHS: Los Angeles County Department of Health Services
LEP: limited English proficient
SFHN: San Francisco Health Network
